# 

**Published:** 1782

**Authors:** 


### P R O M O T E D.

Lately, Dr. Devereux Mytton and Dr. John Matthews to be Candidates, and Dr. Gilbert Blane, Dr. John Whitehead, and Dr. William Lister, to be Licentiates of the Royal College of Physicians in London.

*Sept.* 19. Mr. Mills to be apothecary to the Infirmary at Gloucester, in the room of Mr. Trye.

*Oct.* 26. Mr. Houlston Harries to be surgeon to the forces in Barbadoes.

*Nov.* 2. Dr. Burgoyne Tomkyns to be physician to the Tower of London, in the room of Dr. Robert Petrie.—9. Mr. John Adams to be surgeon to the 13th reg. of foot.—12. Mr. Colin Anderson to be surgeon to the 15th reg. of foot.

*Dec.* 7. William Paine, M. D. member of the College of Physicians, London, to be physician cian to the General Hoſpital in North America. —20. William Fordyce, M.D. phyſician in London, to the rank of knighthood.

### D I E D.

Lately, at Paris, aged 53 years, M. Touſſaint Bordenave, Regius Profeſſor of Surgery in the Academy of Surgery, and member of the Academies of Sciences at Paris, Rouen, and Florence. He was born at Paris April 10, 1728, and was the firſt ſurgeon who filled the office of Sheriff *(Echevin)* of that city. The birth of a Dauphin in 1781, while he held this poſt, was the occaſion of his being created a knight of the order of St. Michael. His death was occaſioned by a paralytic ſtroke, which carried him off in about eight days. He was the author of "Remarques ſur l'inſenſibilité de quelques parties," 12mo. Paris, 1757; "Elemens de Phyſiologie," 12mo. ibid; "Memoire ſur les antiſeptiques," 8vo. ibid. 1769; "Memoire ſur le danger des cauſtiques pour la cure radicale des hernies," 12mo. ibid. 1774; and of ſeveral papers in the Memoirs of the Academies of Surgery and Sciences. (See our 2d vol. p. 182.)—At Marſeilles, aged 84, M. Aubert, M. D. formerly phyſician to the Galleys. During his life-time he gave a ſum of money, equal to about a thouſand pounds ſterling, as a fund for a ſalary to be annexed to the place of phyſician to the hoſpital of the Holy Ghoſt at Marſeilles. He was likewiſe the firſt and principal promoter of the hoſpital for Incurables in that city, in the eſtabliſhment of which he expended five thouſand pounds ſterling. A buſt of this benevolent phyſician is now executing at Paris, and when finiſhed is to be placed in the New Hoſpital, to which he was ſo liberal a benefactor.—At Paris, George de la Faye, a celebrated member of the Academy of Surgery in that city. He was the author of ſeveral papers in the Memoirs of the Academy, and likewiſe of a work, entitled "Principes de Chirurgie", 12mo. the firſt edition of which was printed at Paris in 1739, and the 7th and laſt in 1773. He was alſo the editor of Dionis's Operations of Surgery, with notes; a work which has gone through ſeveral editions.—At St. Lucia, Mr. Thomas Bridge, ſurgeon.

*May* 22, 1782. At Wittemberg, aged 88 years, Daniel William Triller, M. D. Prof. Senior Prof. in the Univerſity at that place.

*Sept.* At Windſor, Mr. William Kimber, apothecary—5. At Cambridge, Mr. Prince, ſurgeon and apothecary.—29. At Glouceſter, Mr. John Matthews, formerly an apothecary of that city.

*Oct.* 9. In Dartmouth-ſtreet, Weſtminſter, Mr. Daniel Lewelin, apothecary.—12. At Taviſtock in Devonſhire, advanced in years, Dr. Lavington, author of a paper printed in the Phil. Tranſ. for 1765, containing the caſe of a young lady, who died of a ſcurvy brought on by the drinking of ſea water.—13. In his 73d year, Edw. Wallis, M. D. an Extra Licentiate of the College of Phyſicians in London, and one of the phyſicians to the Infirmary at York, of of which city he was an Alderman. He ſerved the office of Lord Mayor in 1771.—17. In Thames-ſtreet, London, Mr. Mackay, apothecary.—27. Mr. John Ward, apothecary to the County Hoſpital at Northampton.—31. At Coventry, Mr. Edw. Harpur, jun. apothecary.

*Nov.* 1. Dr. Alexander Ballantyne, phyſician at Saliſbury.—12. At Briſtol, Mr. W. M'Donald, formerly a ſurgeon and apothecary in Jamaica.—14. In conſequence of a fall from his horſe a few days before, Mr. Bathurſt, apothecary in Southwark.—17. At Iſlington, aged 30 years, Mr. Francis Smyth, ſurgeon.—18. At Liverpool, aged 66, Mr. James King, ſurgeon and apothecary.—21. Mr. Thomas Saltonſtall, apothecary in Chapel-ſtreet, Bedford-row, London.

*Dec.* 11. At Braintree in Eſſex, aged 76, Dr. Colin Hoſſack, phyſician to his late R. H. Frederick Prince of Wales, and author of an Abridgement of Van Swieten's Commentaries, in 5 volumes, 8vo.

---

### SECTION IV.

### Quarterly Catalogue.

I. AN inquiry into the nature of the venereal poiſon, and the remedies made uſe of to prevent its effects, principally with reſpect to lotions, pomades, and injections; addreſſed particularly to young men. By *J. Clubbe*, ſurgeon at Ipſwich. 8vo. *Longman*, London, 1782. 2s.

This appears to be the production of a candid and judicious writer. He is of opinion, that we ought ought never to attempt the cure of any ſpecies of venereal affection without the internal uſe of mercury.

2. A letter addreſſed to Dr. Stevenſon, of Newark, occaſioned by a poſtſcript publiſhed in the ſecond edition of his Medical Caſes, with remarks on four letters, written by Philip Thickneſſe, Eſq. By *Edward Harriſon*, member of the Royal Medical Society at Edinburgh. 8vo. *Brown*. London, 1782. 1 s.

3. A medical and philoſophical Eſſay on the theory of the gout. 8vo. *Elmſly*, London, 1782.

4. An epiſtle to Dr. Falconer of Bath. By *Philip Thickneſſe*. 8vo. *Dodſley*, London, 1782.

Dr. Falconer, in his ingenious eſſay on the Bath waters, mentioned a fact which tended to prove that theſe waters are ſlightly impregnated with lead. It ſeems that in 1770, in cleaning the ciſtern of the King's bath, a piece of the upper part of the ciſtern was accidentally broken off, and, on being examined by Dr. Falconer and others, appeared to afford marks of eroſion on its internal ſurface. This fact and Dr. Falconer's concluſions from it are controverted by Mr. Thickneſſe in the preſent pamphlet, in a ſtyle which ſeems to have been dictated by perſonal reſentment.

5. Short ſtrictures on the method of treatment recommended by Dr. Dawſon, in the acute Rheumatiſm. By *Thomas Sanden*, M.D. 12mo. *Payne*, London, 1782. 1 s.

Theſe ſtrictures are well deſerving the attention of the medical practitioner. Dr. Sanden very properly points out the bad effects which may may be expected from the use of large doses of volatile tincture of guaiacum (the remedy recommended by Dr. Dawson) in cases of inflammatory diathesis. An instance is related in which it brought on symptoms of peripneumony, that terminated fatally notwithstanding the repeated use of the lancet.

6. An historical sketch of Medicine and Surgery from their origin to the present time; and of the principal authors, discoveries, improvements, and errors. By *W. Black*, M. D. 8vo. *Johnson*, London, 1782. 315 pages. 5 s.

7. Elements of the theory and practice of Medicine and Surgery. By *John Aitkin*, M. D. 2 vols. 8vo. *Cadell*, London, 1782. 14 s.

8. Verhandelingen van het Batavinasch genootschap der Kansten en Wetenschapen; *i. e.* Memoirs of the Academy of Arts and Sciences at Batavia. 8vo. Batavia. 457 pages.

This, which is the first volume published by the Academy, contains only two papers that will be interesting to the medical reader. In one of these Mr. Van de Steege relates that the small-pox is not more fatal to the Europeans in Java than in their own country; but that it makes great ravages among the negroes, owing to the mode of treatment. He has introduced inoculation with success. In the other paper Mr. Joseph Van Iperen gives an account of a white negro, of the island of Bali, who (as well as four others that are to be described in the second volume by Mr. Valkenaar) is strong, healthy, and five feet high. Mr. Valkenaar's four have produced black children. This induces the author author to doubt the exiſtence of entire families of white negroes.

9. Diſſertatio Phyſica de Aqua. Auctore *Chriſt. Fred. Parrot.* 4to. Erlangen, 1781. p. 30.

10. Tentamen Phyſiologicum de motibus voluntariis. Auctore *Claudio Franciſc. Blaize.* 4to. Nancy, 1782.

11. Der Arzt fur liebhaber der Schoenheit; *i. e.* The Phyſician for the Lovers of Beauty. 8vo. Heidelberg, 1781.

12. *Chriſtiani Theophili Kraſzeinteinii*, Prof. Phyſices in Univerſitate Hafnienſi, Tentamen reſolvendi problema ab Academia Petropolitana propoſitum: qualis ſit natura et character ſonorum litterarum vocalium, a, e, i, o, u; an conſtrui queant inſtrumenta quæ ſonos illos exprimant. 4to. Petropol. 1780.

This eſſay, which obtained the prize from the Academy of Peterſburgh in 1780 (ſee our 2d vol. p. 274), contains a deſcription of the machines invented by the author to expreſs the ſound of the human voice, and a diſcuſſion of the means Nature employs to produce in our mouths the ſound of each vowel.

13. Diſſertatio Inauguralis de Sterilitate humana. Auctore *Baſil. Klutſchatew.* 4to. Fribourg, 1781. p. 41.

The author of this theſis, who is a native of Moſcow, obſerves, that in Ruſſia there are vaſt tracts of country without inhabitants, and numerous marriages without iſſue. He inquires into the cauſe of this national misfortune, and attributes the ſterility of the lower order of women in Ruſſia to their abuſe of ſpirituous liquors.

14. Lettre de la Société Royale de Londres à *M. Herbiniaux*, chirurgien accoucheur et lithotomiste a Bruxelles, au sujet de son traité sur divers accouchemens laborieux et sur les polypes de la matrice. Avec un extrait du Journal de Medecine de la meme ville de Londres, relatif à l'approbation que cet ouvrage a merité, le tout traduit de l'Anglois par *M. Johns*, traducteur fermenté; *i. e.* A Letter from the Royal Society of London to *M. Herbiniaux*, surgeon, &c. at Brussels, concerning his treatise on different laborious deliveries, &c. with an extract from the London Medical Journal relative to the approbation which that work has merited. The whole translated from the English by *M. Johns*, sworn translator. 8vo. Brussels. 1782.

M. Herbiniaux presented a copy of his work to the Royal Society, and in return received the usual compliment of a printed letter of thanks from their secretary. It is this letter, of four lines, which is introduced with so much pomp to the world in the present publication.

If M. Herbiniaux will turn to any of the late volumes of the Philosophical Transf. he will find an advertisement, setting forth, that the thanks given from the chair to persons who present books or other things to the Society, are to be considered in no other light than as a matter of civility; and that when such persons "take the liberty to "report, that they have met with the highest "applause and approbation, it is hoped that no "regard will be paid to such assertions."

In order to enhance the importance of his work M. Herbiniaux (certainly without any foundafoundation in truth) tells his readers, that "several "celebrated physicians, members of the Royal "Society, have condescended to publish, as a "testimony of the *general approbation of the College of Physicians*, a long account of his work, "of which account he has thought it right to "give a *literal* translation." Then follows a translation (but not a *literal* one, or such as might be expected from a *sworn* translator) of what we said of his first volume. Towards the conclusion, where we gave our opinion of his performance, we find our expressions shamefully mutilated. We allowed (*page* 170) that he displayed *considerable* knowledge of his subject; but, instead of *considerable*, he has thought proper to substitute the word *perfect*; and where we observed, that the forceps and lever are "doubtless in *some few cases useful*, but that either of "them will require more practice, to become "expert in handling them, than almost any one "will obtain, who does not frequently have re-"course to them *unnecessarily*." He makes us say, that "in certain cases these two instru-"ments are doubtless *very necessary*, but that "both of them require long practice to become "expert in handling them; an experience which "can be acquired only by the necessity of having "recourse to them *often*." The idea we meant to convey to our readers in the foregoing passage was, that the lever, like the forceps, might do a great deal of mischief, by being often used unnecessarily; and that in midwifery, instruments are not so often required as some practitioners imagine. All this is very artfully metamorphosed to his own advantage by M. Herbiniaux.

### I N D E X.

A.

Page

ACHARD, F. C. chemifch phyfifche fchriften, — 220

Achillis tendo, divifion of, — — 19

Acid, vitriolic, in a native concrete ftate, — — 44

Acrel, Olaus, chirurgical cafes, — — 1, 144

Aitkin, Dr. J. elements of medicine and furgery, — 439

Alum, large dofes of it taken with impunity, — 256

Andry, M. on the hydrophobia, — — 288

Aneurifms, cautions relative to, — — 17

Angina pectoris, new remedy for, — — 308

Arfenic, its efficacy in cancers, — — — 146

Arzt fur liebhaber der fchoenheit, — — 440

B.

Baldaffari, Jof. of a native concrete vitriolic acid, — 44

Bark, Peruvian, condemned, — — 182

——————— Red, obfervations on, — — 248, 313, 432

Beau, J. F. le, eulogy of, — — — 157

Belladonna, its efficacy in canine madnefs, — — 199

Berger, Mr. on faltpetre, — — 22

Bergius, Dr. P. on foap and lime water in calculus, — 24

Bergman, Thorb. letter from, — — — 89

——————— opufcula phyfica et chemica, — 331

Beffi, L. parere medico-legale, — — — 112

Beunie, M. de, on the poifonous effects of mufcles, — 134

Bertholon, Abbé, electricité du corps humain, — — 215

Bianconi, J. L. lettere fopra A. C. Celfo, — — 217

Birds, receptacles of air in injected, — — 198

Black, Dr. W. hiftorical fketch of medicine, — — 439

Blaize, C. F. de motibus voluntariis, — — 440

Bland, Dr. Robert, his midwifery tables, — — 113

Bloodletting, miftakes of the ancients relative to, — 264

Bones, their fenfibility in a difeafed ftate, — — 223

Born, Ignat. index mufei Cæfarei, — — 220

Brain, difeafes of, — — — 4, 146

Brandelius, L. de fenfibilitate offium, — — 223

Breuchmann, F J. de morbis nervorum, — — 110

Broc, G. de pleuritide, — — — 332

Broughton, Dr. A. on the catarrh of 1782, — — 297

Brouffonet, P. M. A. Ichthyologia, — — 326

Bruffells, memoirs of the Academy at, — — 133

Burning glafs, its ufe in furgery, — — — 274

C.

Calculus, obfervations on, — — 16, 24

Calomel, new mode of preparing it, — — 283

Caluri, F. his account of an hermaphrodite, — — 44

Camper, Dr. Peter, obfervations by, — — 356, 432

Cancer, inftances of, — — — 4, 5, 6, 146

Carlifle, account of the jail fever at, — — 268

L l l 2 Carrere,

### I N D E X.

Page

Carrere, M. fur la douce amere, 111
Caftration, for the cure of hernias, condemned, 273
Catalepfy, cafes of, 144, 376
Cataract, remarks on, 7
Catarrhs, epidemic, remarks on, 152, 202, 294, 297, 318
Cautery, actual, origin of, 262
Chefter, report of the fmall-pox fociety at, 427
Clennachen, Geo. M. de vino, 213
Clubbe, J. on the venereal poifon, 437
Cold, powers that animals have of producing it, 122
Cooke, Captain, the healthinefs of his crew inftanced, 48
Crawford, Dr. Adair, on the power of animals to generate cold, 122
Crinons, an account of, 289
Cupping, antiquity of, 266

D.

Darbey, Mr. R. on the ufe of cod liver oil, 398

David, M. fur le necrofe, 369

Dawfon, Dr. Tho. his remedy for fore eyes, 180

Deaths, 100, 210, 320, 435

Debreft, M. fur les eaux de Bourbonnois, 111

Delius, J. des mohnfaffts in der Luftfeuche, 221

Digeftion, experiments on, 309

Duhamel, M. traité des peches, 112

Dujardin, M. hiftoire de la chirurgie, 260

Duncan, Dr. Andrew, letter to Dr. Jones, 104

Durande, M. elemens de botanique, 335

Dyfentery, cured by nux vomica, 189

E.

Eggs, incubation of by electricity, 221

Electricity, medical, new thoughts on, 327

Elliot, Dr. J. elements of natural philofophy, 59

Elfe, Jofeph, works of, 380

Encyclopedie, profpectus of, 200

Epidemics, obfervations on, 152, 286, 287, 288

Eye, fore, remedy for, 180

—— orbit of, tumour extracted from, 6

—— cancerous, fuccefsfully extirpated, ibid.

F.

Falconer, Dr. W. on the epidemic catarrh, 294

Fever, putrid, obfervations on, 45, 208

—— puerperal, improved mode of treating it, 411

Fink, S. L. de morbis biliofis anomalis, 217

Fire, St. Anthony's, inquiries relative to, 353

Ford, Edward, of an ulceration of the bladder, 80

Fothergill, Dr. John, life of by Dr. Thompfon, 215

—— Dr. Anthony, hints on animation, 185

—— on the puerperal fever, 411

Fracture of the legs, new machine for, 334

Furor uterinus, how cured by Mofchion, 343

Gmelin,

### INDEX.

G. Page

Gmelin, J. F. einleitung in die pharmacie, — — 332

Gout, essay on the theory of, — — — 438

Gum elastic, experiments relative to, — — 221

H.

Hahn, Dr. J. D. letters from to Dr. Simmons, — 313, 431

Hair powder, dissertation on, — — 217

Hagstrom, J. O. on the use of nux vomica in dysentery, — 189

Haller, Alb. supplement to his physiology, — — 220

Hamilton, Dr. R. on the influenza, — — 213

Harrison, Gul. de lue venerea, — — — 214

——— Edward, letter to Dr. Stevenson, — — 438

Hawes, Dr. W. his address to the Parliament, — 184

Head, peculiar tumour on, — — — 4, 146

Henry, Thomas, on the medicinal effects of magnetism, — 303

Herbiniaux, G. œuvres de, — — 161, 242, 441

Hermaphrodite, instance of, — — — 44

Hernias, remarks on, — — — 12, 273

Heysham, Dr. John, on the jail fever at Carlisle, — 268

Home, Jacobus, de scorbuto, — — 213

Homer, his medical knowledge, — — — 264

Howard, John, on the medical properties of mercury, — 170

Hydrocele, observations on, — — 16, 144, 381

Hydrocephalus internus, cured by blisters, — — 402

Hydrophobia, observations relative to, — 23, 32, 288, 307, 312

I.

Jamaica, observations on the climate and diseases of, — 63

Jaubert, M. on exanthematous fevers, — — 362

Jebb, Dr. John, on the palsy of the lower limbs, — 372

Ingenhousz, J. experiences sur les vegetaux, — — 110

Johnstone, Joh. de apoplexia, — — — 214

Jones, Dr. Robert, on the state of medicine, — 104, 328

K.

Kite, Charles, cases by, — — — 306, 405

Klutschatew, B. de sterilitate humana, — — 440

Kostlini, C. H. fascic. animadversionum, — — 218

Kranzeintein, C. T. de natura, &c. vocalium, — 440

L.

Landais, M. sur l'allaitement des enfans, — — 224

Lapland, Swedish, population of, — — 22

Launay, M. de, on the orichalcum of the ancients, — 141

Lee, Dr. John, of a gouty case, — — 107

Leech, medicinal, natural history of, — — — 139

Leprosy, account of at Martigues, — — 289

Levelingii, H. P. obs. anatom. rariores, — — 216

Lever, of Roonhuysen, description of, — — 165

Lindsay, A. de plantarum incrementi causis, — — 214

Linnæus, view of his life and writings, — — 29

Lister, G. de fermentatione, — — 213

Lithotomy, remarks on, — — — 16

Loadstone,

Page

Loadſtone, its medicinal effects, — — 158, 303

London, comparative table of its population, — 120

Lorry, C. ſur les alimens, — — — 111

M.

Magneſia, calcined, experiments on, — — 96

Mancheſter, philoſophical ſociety at, — — 95

Marci, Abbé, on the tinning of culinary veſſels, — — 141

Martin, R. on the external ſenſes, — — 20

——— his ſtatical experiments, — — — 147

Meaſles, treatment of by bliſters, — — — 183

Membrana caduca, Aretæus ſuppoſed to allude to it, — 341

Mercury, treatiſe on, — — — 170

Metzer, M. gerichlich medinifche beobachtungen, — 332

Milman, Dr. F. on the origin of ſcurvy, — — 45

Mönch, M. his expts. on calcined magneſia, — — 96

Mongez, M. of a machine for fractured legs, — — 333

Murray, A. de ſenſibilitate oſſium, — — 222

——— J. A. de limitanda laude librorum, — — 224

Muſhrooms, their noxious qualities, — — 362

N.

Necroſis, obſervations on, — — — 369

Needham, Abbé, on horned cattle, — — 135

Nicolas, M. des eaux minerales de Lorraine, — — 110

Nux vomica, recommended in dyſentery, — — 189

O.

Odhelius, J. L. caſes and obſervations by, — 23, 147

Oeſophagus, abſceſs of, — — — 157

Opium, its effects in lues venerea, — — — 221

——— new method of preparing an extract of, — — 283

——— obſervations on its analyſis, — — 360

Orichalcum, of the ancients, what, — — — 141

Oxford, ſchool of phyſic at, — — — 422

P.

Palſy of the lower limbs, remarks on, — 225, 372, 405

Paris, memoirs of the Royal Medical Society at, — 149, 273, 353

Paronychia, a particular kind of, — — 17

Parotids, extirpation of, — — — 8

Parrot, C. F. de aqua, — — — 440

Percival, Dr. on the uſe of cod liver oil in rheumatiſm, — 392

Perkins, Dr. on epidemic catarrhs, — — — 152

Peyrilhe, M. hiſtoire de la chirurgie, — — 337

Pharmacopœia, pauperum, Hamburg, — — 334

Phlogiſton, its nature aſcertained, — — — 88

Phthiſis, pulmonalis, eſſay on, — — 385

Plague, cured by icy frictions, — — — 329

Plants, catalogue of at Hohenheim, — — — 332

Platina, its fuſibility, — — — 308

Polypus, of the noſe, remarks on, — — — 7

——— uterine, treatiſe on, — — — 242

Pott, Percival, on the palſy of the lower limbs, — — 225

Price,

Page

Price, Dr. James, experiments on mercury, ſilver and gold, — 328

Prognoſtic, in acute diſeaſes, obſervations on, — 257

Promotions, — — — 99, 209, 319, 434

Pubis, oſſa, ſeparation of in parturition, — — 14, 278

Putridity, in diſeaſe, a vague term, — — 46

Q.

Queſtions, prize, — 85, 87, 196, 197, 198, 305, 306, 419

R.

Rancé, Don Juan, de materia medica, — — 385

Rectum, deficiency of, — — — 26

Reid, Dr. T. on phthiſis pulmonalis, — — 385

Reuſs, C. F. on hair powder, — — — 217

Rheumatiſm, cured by cod liver oil, — — 392

Rigler, N. de conſtitut. epid. ann. 1775, &c. — — 332

Rondeau, M. du, on the poiſon of muſcles, — — 135

———————— medicinal leech, — — 139

Ronnou, M. of arſenic in cancers, — — 146

Roy, Charles le, on prognoſtics, — — — 257

Ryan, Dr. Dennis, on remittent fevers, — — 63

Rye, ſmutty, its effects, — — 353, 361

S.

Saltpetre, how prepared at Helſingfors, — — 22

Samoilowitz, Dr. on the cure of the plague, — — 329

Sammlungen zur phyſik und naturgeſchichte, — — 219

Sanden, Dr. T. ſtrictures on Dr. Dawſon, — — 438

Sarcocele, ſingular caſe of, — — — 426

Saunders, Dr. W. on the red Peruvian bark, — — 248

Schœpff, J. D. des mohnſaffts in der Luſtſeuche, — 221

Schweder, C. F. auſerleſne Vollſtœndige, &c. — — 335

Schotte, Dr. ſingular caſe of ſarcocele, — — 426

Scurvy, obſervations relative to, — — — 45

Section of the ſymphyſis pubis, obſervations on, — 31, 366

Selle, C. G. medicina clinica, — — — 334

Sheep, method of bleeding them, — — — 355

Sienna, atti dell'academia di, — — — 43

Simmons, Dr. letters to, — 80, 300, 303, 313, 405, 431, 432

Small-pox, in a fœtus in utero, — — — 121

Soaps, acid, remarks on, — — — 359

Societatis, Jablonovianæ, acta, — — — 334

Soinne, J. le, de thermis aquis granenſibus, — — 335

Soliva, Salvador, ſobre el ſen de Eſpana, — — 336

Spalanzani, Abbé, his experiments on digeſtion, — 309

Spangerberg, G. obſ. obſtetric. decas, — — 333

Stark, Dr. his account of tubercles, — — — 387

Stevenſon, Dr. W. caſes in medicine, — — 182

Stockholm, memoirs of the Academy of, — 20, 142

Stuart. Car. de ſyſtemate nervoſo, — — — 213

Surgery, hiſtory of, — — — 269, 337

Sweden, obſervations on its climate, — — — 142

Swediar, Dr. F. of a fungus of the bladder, — — 190

Table,

Page

T.

Table, meteorological, for 1780, — 84

Tænia, method of treating it, — 157

Tartar, emetic, improved modes of preparing it, — 358, 420

Teeth, obſ. on their growth, — 221, 364

Thickneſſe, Philip, his letter to Dr. Falconer, — 438

Thompſon, Dr. G. his life of Dr. Fothergill, — 214

Thunberg, C. P. on bezoar, — 143

Torre, Padre della, on the blood, — 218

Tranſactions, Medical, German tranſlation of, — 216

—— Philoſophical, vol. 71, — 113

Tranſmutation of metals, expts. relative to, — 328

Trichiaſis, its cauſe and how removed, — 5

Tubercles, of the lungs, how formed, — 387

Tumours, encyſted, how to be treated, — 10

U.

Vagina, cloſed by a thick membrane, — 159

Vaux, Geo. his edition of Mr. Elſe's works, — 380

Verhandelingen van het Batavinaſch &c. — 439

Vicq D'Azyr, M. expts. and obſ. by, — 156, 357

Viper, effects of its bite, — 145

Virus, venereal, experiments on, — 214

Vomica, caſes of, — 11

Urethra, fiſtulas of, not always venereal, — 14

Uterus, double, inſtances of, — 19, 425

—— retroverted, known to the ancients, — 348

W.

Walter, J. G. de ſectione ſymphyſis, — 366

Waters, mineral, works relative to, 110, 111, 134, 284, 327, 360, 361

Wenſels, C. F. chemical eſſays, — 215

White, Dr. R. caſe of hydrocephalus, — 402

Wiedenfeld, O. P. de vulnerum medela, — 223

Willan, Dr. R. on the Croft water, — 327

—— letter to, — 430

Winterbottom, Joh. de vaſis abſorbentibus, — 214

Wintringham, Clifton, de morbis quibuſdam, — 177

Wiſilikally, G. de lue venerea, — 216

Wood, Dr. Loftus, valetudinarian's companion, — 213

Worms in the meatus auditorius, caſe of, — 9

Wriſberg, H. A. de ani defectu, — 26

X.

Xiorro, J. G. aphoriſmos de Boerhaave, — 216

